# Supplementary material for: Identification of photoperiod-induced specific miRNAs in the adrenal glands of Sunite sheep (Ovis aries)
Source: Front Vet Sci. 2022 Jul 22;9:888207. doi: 10.3389/fvets.2022.888207 (PMC9354845; doi:10.3389/fvets.2022.888207)
Supplement: Supplementary file 1 [file Data_Sheet_1.ZIP › Supplementary file/miRDeep2 file.zip!/miRDeep2 file/Novel_Structure/SP42A1/Novel_72.pdf]

Provisional ID	:	NC_019463.2_10592
Score total	:	28.7
Score for star read(s)	:	3.9
Score for read counts	:	21.5
Score for mfe	:	1.7
Score for randfold	:	1.6
Score for cons. seed	:	
Total read count	:	54
Mature read count	:	52
Loop read count	:	0
Star read count	:	2

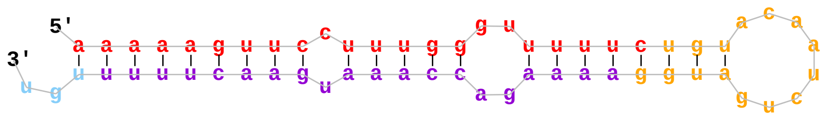

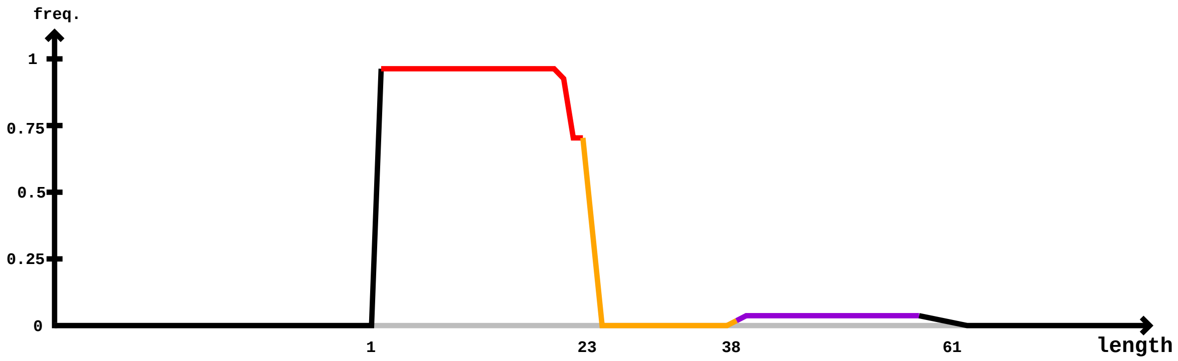

Mature Star

```
5'- aauaucuauuucaaguaucuauuggguuggacaaaaaguuccuuuggguuuuucuguacaaucugauggaaaagaccaaaugaacuuuuuguuaaauccaauauuuauucau -3'  obs
    aauaucuauuucaaguaucuauuggguuggacaaaaaguuccuuuggguuuuucuguacaaucugauggaaaagaccaaaugaacuuuuuguuaaauccaauauuuauucau       exp
    .............((((..(((((((((.((((((((((((.(((((..((((((((........))))))))..))))).))))))))))))).)))))))))..))))...     reads   mm   sample
    ................................aaaaaguucGuuuggguuu.............................................................     2       1    seq
    ................................aaaaaguucGuuuggguuuu............................................................     12      1    seq
    ................................aaaaaguucGuuuggguuuuuc..........................................................     38      1    seq
    .....................................................................aaaaAaccaaaugaacuuuu.......................     1       1    seq
    ......................................................................aaagaccaaaugaacuuuuugu....................     1       0    seq
```
